# Transnational migration and Mexican women who remain behind: An intersectional approach

**DOI:** 10.1371/journal.pone.0238525

**Published:** 2020-09-14

**Authors:** Higinio Fernández-Sánchez

**Affiliations:** 1 Faculty of Nursing, University of Alberta, Edmonton, Alberta, Canada; 2 Edmonton Clinic Health Academy, University of Alberta, Edmonton, Alberta, Canada; USC Keck School of Medicine, Institute for Global Health, UNITED STATES

## Abstract

**Objective:**

To explore the scope, range, and nature of the existing literature on Mexican women who remain behind in their communities of origin while their partners migrate abroad.

**Design:**

A scoping review informed by an intersectionality framework was conducted over four months, January-April 2020.

**Data sources:**

The electronic databases Medline, PsyINFO, Global Health, CINAHL, Gender Studies Database, Dissertations & Theses Global, LILACS, IBECS, and Sociological Abstracts were searched.

**Review methods:**

Articles were included if they focused on Mexican women who remain behind across transnational spaces. Two independent reviewers screened and selected articles. Data were analyzed and synthesized using descriptive statistics for quantitative data and content analysis for qualitative data.

**Results:**

A total of 19 articles were included for analysis; within those, the methods used included quantitative (n = 5), qualitative (n = 11), mixed methods (n = 2), and intervention (n = 1). Most studies lacked a theoretical framework (n = 10); the majority were empirical published studies (n = 11), and most used interviews (n = 12) and surveys (n = 6) to collect data. All of the articles studied cis-heterosexual Mexican women. Major areas identified were 1) research context, 2) gender roles, and 3) women’s health.

**Conclusion:**

Implications for practice and future research are discussed.

## Introduction

As the world continues to interconnect at rapid rates, transnational migration has become the center of attention for many governments worldwide, in particular those in North America. Transnational migration is defined as “a process of movement and settlement across international borders in which individuals maintain or build multiple networks of connection to their country of origin while at the same time settling in a new country” [1]. Historically, transmigrants, or individuals who migrate across international borders, maintain strong ties with their countries of origin by constant involvement with the social, political, and cultural systems within these countries [1].

The International Organization for Migration reported nearly 272 million international migrants in 2019 [2]. Latin America and the Caribbean were among the highest regions of origin of these migrants (37.7 million), most of whom were men (51.6%). The leading destinations were European countries (with 58 million migrants), followed by the United States and Canada (with 57.7 million migrants combined). In the case of Mexico, according to the United Nations, more than 11 million Mexicans resided in the United States in 2017 [3] and over 400,000 Mexicans entered Canada in 2018 [4]. Even though the number of Mexican women migrating has also been on the rise, the vast majority of migrants are men who have left their families behind.

The increasingly militarized border between the United States and Mexico has lengthened migrant stays in the United States [5]. This situation often prevents migrants from making more frequent visits to their families in Mexico, thus restructuring their family dynamics. According to one study [6], even when living conditions of families who remain behind have been seen to improve with the money remittances provided by these men, the burden is often added these families in their originating communities. For instance, if Mexican migrants reach the United States, immediate job offers may not be available to them, thus preventing them from economically supporting their families [7].

A recently published work on women who remain behind across international spaces shed light on two major areas: 1) women’s health; and 2) women’s social, economic, and cultural conditions [8]. Many mental health concerns, like feelings of loneliness and sadness, stress, and anxiety, are a result of the migrant partner’s absence. The appearance of new roles and responsibilities, the change in family structure, and the relationship maintenance between the couple have been linked to the increase in burden for women who remain behind. Changes in women’s sexual and reproductive well-being are also noticed. For instance, growth in women’s autonomy and empowerment has shown to improve sexual practices, such as using a condom. Moreover, women have better access to healthcare due to the remittances they receive from their migrant partners.

Despite the growing body of literature on women who remain behind while their partners cross transnational borders, an intersectional approach has not been used. Due to the long-term binational migration pattern between the United States and Mexico, it is important to further explore the literature on Mexican women who remain behind through an intersectional lens to address this gap in the literature. My research has the following objectives: 1) to locate the empirical evidence on Mexican women who remain behind (MWRB) in the context of transnational migration; 2) to analyze and synthesize literature on this population through an intersectional lens; and 3) to identify and report gaps in the research literature on this topic.

## Theoretical framework

The use of theoretical frameworks in scoping reviews can help us better understand the relationships among variables [9] and can provide a clearer direction for future research areas [10]. Adapting the scoping review methodology with intersectionality theory as a critical framework “can shed light on the experiences of individuals who belong to multiple disadvantaged social groups, such as being black and low income, an immigrant, and/or in poor health” [11, p417]. This critical paradigm can provide ways of understanding how social systems support and preserve inequalities of marginalized groups. Furthermore, it aids in addressing complex inequalities in a way that is meaningful for people.

For this scoping review, I relied on an intersectionality framework [10]. Intersectionality seeks to uncover gaps in the literature by centering on those whose experiences and necessities are visible in the literature and on those whose voices are being silenced. The philosophical underpinnings of the intersectionality are rooted in critical race theory [12] and feminist theory [13], specifically the resistance brought about by Black feminism in the 1960s and 1970s in the United States, and the struggles of “Third World Women” [12, 13]. Intersectionality comprises the exploration of intersecting vectors, such as race, social class, gender, and power, while acknowledging that the combinations of such vectors may influence the way the intersections are experienced [14]. Intersectionality suggests that gender and patriarchy must be explored in all intersectional analyses; hence, this intersectional perspective is embedded in an intersectionality framework. Given the complexity of real-world factors of remaining behind during transnational migration, I considered the IF appropriate to guide this scoping review because it helped me analyze the literature through an intersectional lens.

## Materials and methods

I conducted a systematic literature review from January to April 2020. For this review, the five-step scoping review methodology was employed [15–17]. In contrast to systematic reviews that are largely conducted to explore, appraise, and synthesize research evidence on the effectiveness of randomized controlled trials [18–20], scoping reviews can be used to assess the extent and nature of the existing body of literature to develop a theory or to inform whether a full systematic review is needed [21, 22]. The steps for scoping reviews are 1) identification of the research question(s), establishing inclusion and exclusion criteria, and selecting search terms; 2) identification of relevant studies through a comprehensive and systematic search of the literature in electronic databases; 3) selection of studies; 4) data extraction and charting; and 5) data analysis and synthesis, and communicating the findings.

The question “What is the scope, range, and nature of the existing literature on Mexican women who remain behind while their partners migrate abroad?” guided this scoping review. I combined the search terms from three categories: population, concept, and context [23]. The population for this review is women, and the concept is remaining behind and the context in Mexico. These concepts were adapted to every database searched. Subject headings and keywords from the Medical Subject Headings (MeSH) were used for each of the concepts to retrieve empirical studies. Table 1 shows the search terms included for this review: Left-behind OR remain*-behind OR nonmigrant* OR non-migrant* OR stay*-behind*. These search terms will be combined with female* OR wom?n OR mother* OR spous* OR partner* OR wife OR wives, AND Mexican* OR México OR Aguascalientes OR Baja California OR Baja California Sur OR Campeche OR Chiapas OR Chihuahua OR Ciudad de México OR Coahuila de Zaragoza OR Colima OR Durango OR Estado de México OR Guanajuato OR Guerrero OR Hidalgo OR Jalisco OR Michoacán de Ocampo OR Morelos OR Nayarit OR Nuevo León OR Oaxaca OR Puebla OR Querétaro OR Quintana Roo OR San Luis Potosí OR Sinaloa OR Sonora OR Tabasco OR Tamaulipas OR Tlaxcala OR Veracruz de Ignacio de la Llave OR Yucatán OR Zacatecas.

**Table 1:** Search strategy. Edmonton, AB, Canada, 2020.

**AND**			
**OR**	**Concept 1: Left-behind**	**Concept 2: Population**	**Concept 3: Context**
	left-behind	mother*	Mexican* OR México OR Aguascalientes OR Baja California OR Baja California Sur OR Campeche OR Chiapas OR Chihuahua OR Ciudad de México OR Coahuila de Zaragoza OR Colima OR Durango OR Estado de México OR Guanajuato, Guerrero OR Hidalgo OR Jalisco OR Michoacán de Ocampo OR Morelos OR Nayarit OR Nuevo León OR Oaxaca OR Puebla OR Querétaro OR Quintana Roo OR San Luis Potosí OR Sinaloa OR Sonora OR Tabasco OR Tamaulipas OR Tlaxcala OR Veracruz de Ignacio de la Llave, OR Yucatán Zacatecas.
	remain* behind	spous*	
	stay* behind	wives	
	non-migrant*	wife	
	nonmigrant*	wom?n	
		female*	

Articles were included if they (a) focused on Mexican women who remain behind while their partners migrate abroad; (b) were published empirical studies and grey literature such as unpublished material and dissertations; and (c) had the English or Spanish full texts available. There was not any restriction on the study designs to generate a breadth of coverage on available literature, and other languages were not considered due to the resources involved in having translations done. Studies were not excluded by date limits to explore the literature broadly. Articles were excluded if they focused on left-behind children, parents, or grandparents of migrants, or if they focused on left-behind women in the context of militarization or internal migration, as these topics did not offer an answer to the research question of this scoping review.

Articles were identified through the search of electronic databases. The intersectionality framework guided my choice of databases to include those with gender, health, nursing, social sciences, and Latin American and Caribbean sources. The following databases were explored by a Ph.D. nursing student: Medline, PsyINFO, Global Health, CINAHL, Gender Studies Database, Dissertations & Theses Global, LILACS (Literatura Latinoamericana y del Caribe en Ciencias de la Salud), IBECS (Índice Bibliográfico Español en Ciencias de la Salud), and Sociological Abstracts. To keep track of the articles, those that were identified were exported and organized into RefWorks, a web-based reference manager. Once organized, I imported the articles to Covidence, a web-based software that organizes knowledge synthesis reviews [24].

**Fig 1:**
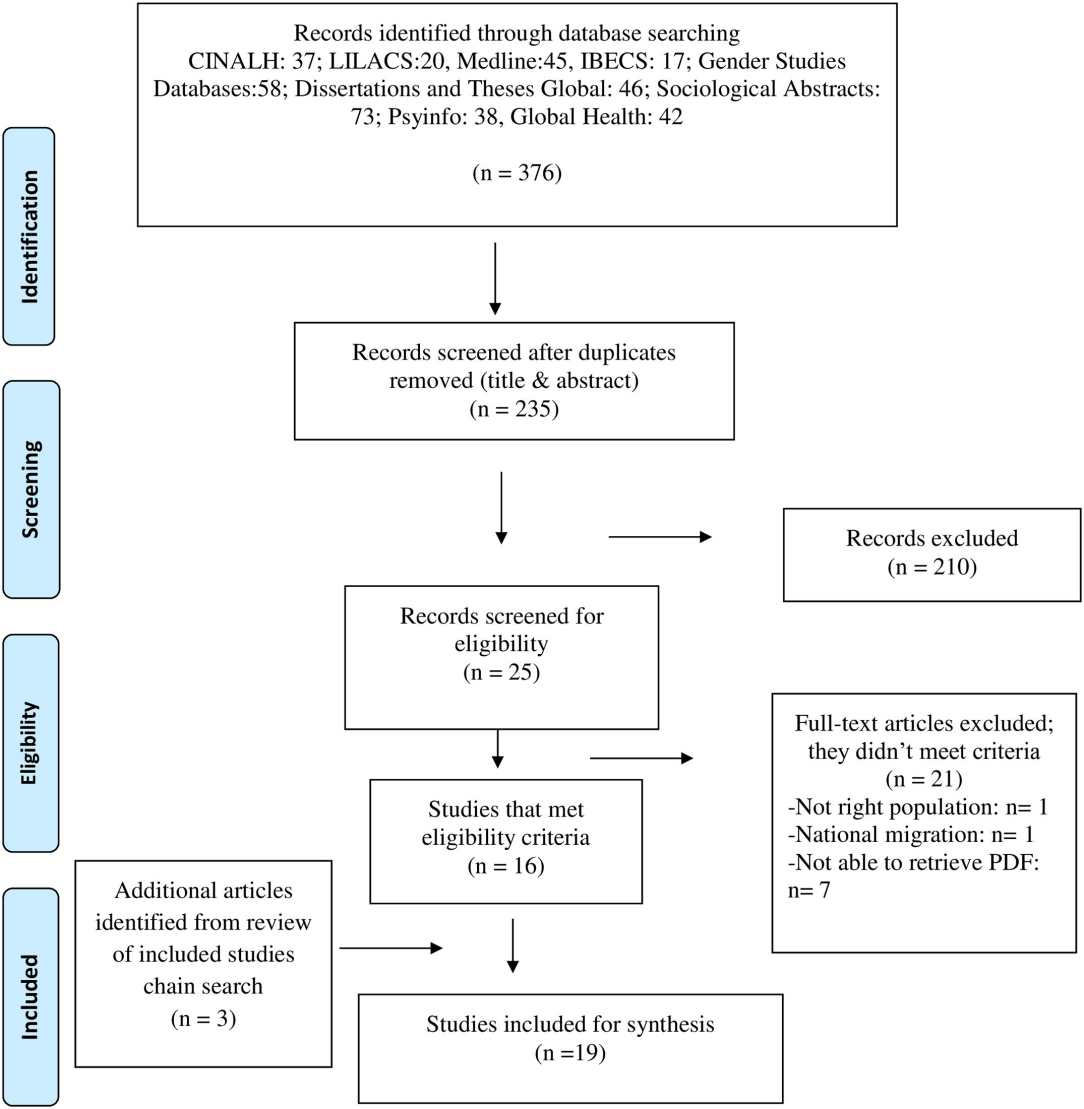
Flow chart. Edmonton, AB, Canada, 2020.

Two independent reviewers screened and selected articles. First, titles and abstracts were screened using a present diagram for the inclusion and exclusion criteria. The remaining articles were then screened full text for eligibility. Lastly, a snowballing technique was employed to systematically hand search the reference lists of the included studies for analysis. This supplemented the search by allowing me to locate relevant studies that met the inclusion criteria of this scoping review that were not spotted during the database search [25]. Fig 1 shows the process for screening and selecting studies for this scoping review [26].

One reviewer extracted and charted the studies’ information, and a second reviewer verified it. Three main pieces of information: publication details (i.e. publication dates); key characteristics of each study (i.e. sample sizes); and summaries of study findings. Data synthesis involved numerical analysis for quantitative data and directed content analysis for qualitative data. For the quantitative data, the Statistical Package for the Social Sciences (SPSS) version 22 for Mac OX was used to aid in the analysis. Qualitative data was managed, analyzed, and explored using QUIRKOS, a qualitative analysis software.

The directed content analysis approach allowed me to a) identify repeating text, b) code the identified text using predetermined codes based on an intersectionality framework (i.e. health), and c) create new codes for the text that could not be categorized within the original coding scheme. Furthermore, a directed content analysis [27] guided by an intersectional lens helped me 1) emphasize the distinction between those whose experiences are being considered in the literature and those whose experiences are not, and 2) contemplate social, political, cultural, and religious implications as intersecting categories. To minimize the risk of bias in this scoping review, a) two independent reviewers conducted data screening, b) conflicts were resolved by consensus, and c) one reviewer extracted data and a second reviewer verified it.

## Results

**Fig 2:**
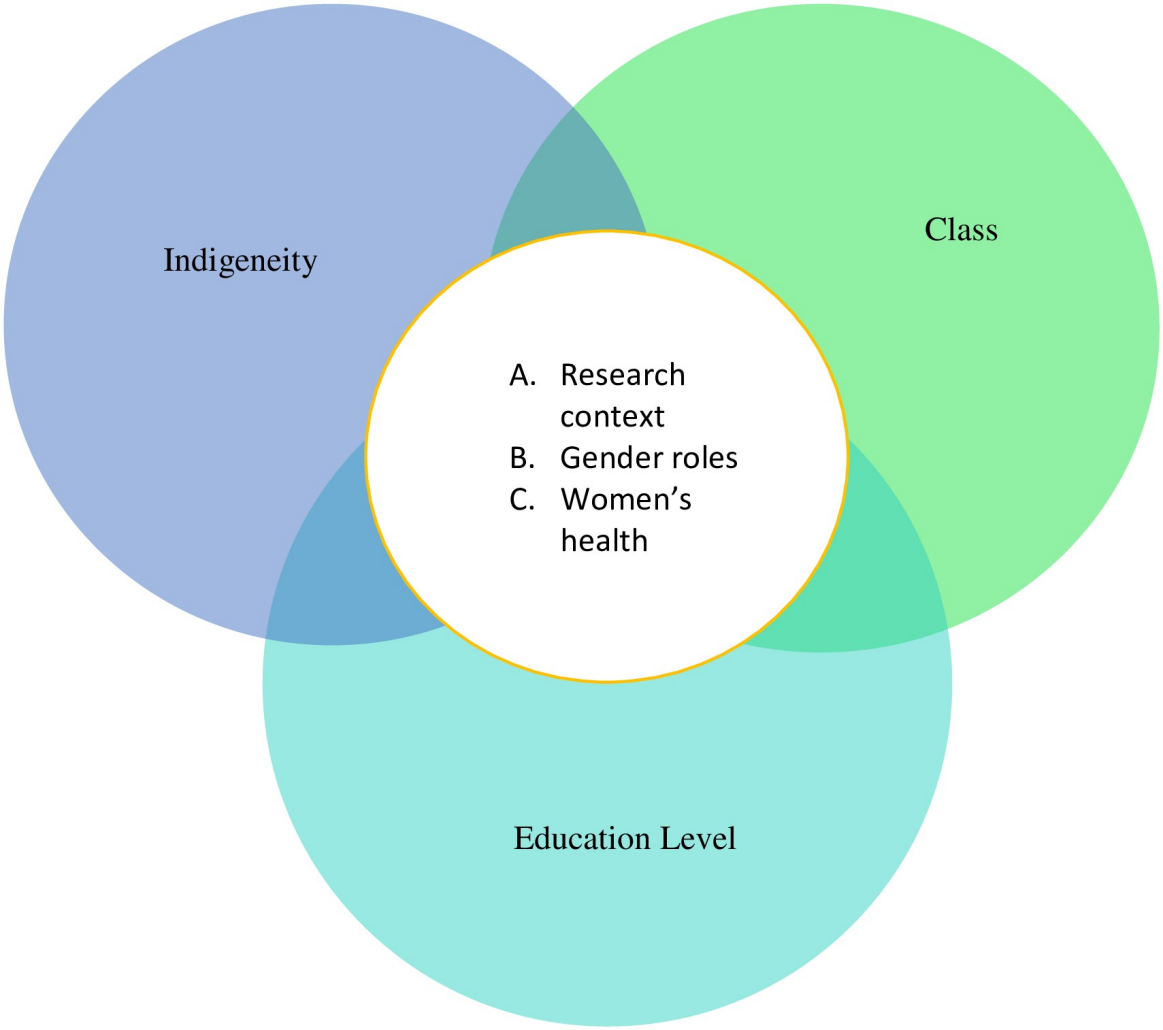
Visible areas across the empirical literature on Mexican women who remain behind. Edmonton, AB, Canada, 2020.

A total of 19 articles were included for analysis; key findings are presented in Tables 2 and 3. In general, researchers used several approaches to their work: quantitative (n = 5), qualitative (n = 11), mixed methods (n = 2), and intervention (n = 1). Most authors relied on theories related to gender, power, migration, stress, and agriculture. Furthermore, the majority of the articles were empirical published studies (n = 12), and the rest were theses and dissertations (n = 7). Interviews and surveys were the primary data collection methods. Through the intersectional analysis, I was able to identify three major areas for discussion: 1) research context, 2) gender roles and patriarchy, and 3) women’s health. These areas were often situated over issues of gender, class, education, indigeneity, and health (Fig 2).

**Table 2:** Study key characteristics. Edmonton, AB, Canada, 2020.

	Year	Source type	Research Design	Research Methods	Sampling	Sample
1	2018 [28]	Empirical study	Quantitative	Surveys	Cross sectional	n = 4657
2	2017 [29]	Thesis	Qualitative	Interviews	Non-Random sampling	n = 9
3	2016 [30]	Dissertation	Qualitative	Interviews	Purposive sampling	n = 5
4	2015 [31]	Empirical study	Quantitative	Surveys	Randomly selected	n = 2813
5	2014 [32]	Thesis	Qualitative	Interviews	Snowball sampling	n = 35
6	2014 [33]	Empirical study	Qualitative	Interviews	Purposive sampling	n = 22
7	2014 [34]	Empirical study	A pilot intervention study	Pre-post design	Snowball sampling	n = 39
8	2013 [35]	Dissertation	Mixed methods	Surveys and interviews	Systematic & Snowball sampling	Surveys (n = 71), interviews (n = 9)
9	2013 [36]	Empirical study	Qualitative	Interviews	-	n = 8
10	2012 [37]	Empirical study	Quantitative	Survey	Randomly selected	n = 1,850
11	2012 [38]	Empirical study	Qualitative	Focus groups, surveys and interviews	Snowball sampling	Focus (n = 6); surveys (n = 14); interview (n = 16)
12	2009 [39]	Empirical Study	Quantitative	Surveys	Probabilistic sampling	n = 418
13	2008 [40]	Empirical study	Qualitative	Interviews	Snowball sampling	n = 60
14	2008 [41]	Dissertation	Qualitative	Interviews and surveys	Purposive, snowball and convenience sampling	Interviews (n = 16), surveys (n = 13)
15	2007 [42]	Dissertation	Qualitative	Interviews	Non-Random sampling	n = 9
16	2007 [43]	Empirical study	Qualitative	Interviews	Snowball sampling	n = 14
17	2002 [44]	Dissertation	Mixed Methods	Interviews	-	-
18	2002 [45]	Empirical study	Qualitative	Interviews	Random Sampling	n = 53
19	1993 [46]	Empirical study	Quantitative	Surveys	Snowball sampling	n = 202

**Table 3:** Study key characteristics. Edmonton, AB, Canada, 2020.

	Year	Location	Migrant’s Destination	Participant’s Age	Migrant’s Gender	Theoretical Framework
1	2018 [28]	Not clearly stated	USA	18–66	Male	Migration and Gender
2	2017 [29]	Guanajuato	USA	25–44	Male	Gendered Global Framework
3	2016 [30]	Puebla	USA	35–45	Male	Gender and Migration Theory
4	2015 [31]	Mexico	USA	18–44	Male	-
5	2014 [32]	Guanajuato	USA	20–75	Male	Transnational migration & Gender Theories.
6	2014 [33]	Yucatán	USA	18–55	Male	Theory of Gender & Power
7	2014 [34]	Guanajuato	USA	27–54	Male	Cognitive Behavioral Theory, Psychoeducation & Social Support
8	2013 [35]	Oaxaca	USA	35–50	Male	Gender Theory
9	2013 [36]	Calvarito, in the State of Mexico	USA	23–60	Male	-
10	2012 [37]	Morelos, Veracruz, Jalisco, Michoacán & Yucatán	USA	-	Male	-
11	2012 [38]	Villanueva, Zacatecas or Campeche	USA	22–48	Male	Transnational migration
12	2009 [39]	Guerrero, Oaxaca & Puebla	USA	15–49	Male	Model of Depression & Stress-Meditation-Outcome Theory
13	2008 [40]	Central Mexico	USA	20–50	Male	-
14	2008 [41]	Villanueva, Zacatecas or Campeche	USA	22–48	Male	Feminization of Agriculture Framework
15	2007 [42]	Central & Southern Coast of Mexico	Canada	-	Male	Structural and Network Theory of Migration
16	2007 [43]	Oaxaca	USA	-	Male	-
17	2002 [44]	Mexico	USA	-	Male	-
18	2002 [45]	Yucatan or Yucatán	USA	40–60	Male	Gender and Migration Theory
19	1993 [46]	Jalisco & Michoacán	USA	26–46	Male	Stress-Meditation-Outcome Theory

### Research context

The analyzed articles in this study focused on some of Mexico’s poorest and most marginalized communities—Guerrero, Oaxaca, Veracruz, Puebla, and Campeche [28, 29, 32, 34, 47]—some of which are high in Indigenous populations [30, 32, 36]. Mexican women represented in this literature have little schooling; the majority have nine years or less of formal education [28, 30, 32, 34, 39, 48]. Additionally, it is important to note that women in these articles respond to cis-heterosexual females, where the male partners assume *breadwinner* titles and migrate to live and work abroad to provide for their families [36, 39, 49, 50] while the women remain to care for their families [48]. Male partners are seen to migrate to high-income countries like the United States and Canada [28–39, 47–53].

### Gender roles and patriarchy

The literature shows that many of the Mexican women who remain behind continue living in patriarchal societies and behave according to traditional gender roles [33, 35, 38, 47, 49]. Patriarchy refers to “a social structure where the actions and ideas of men are dominant over those of women” [54]. Even though some studies report that men migrating transnationally is common and expected in Mexican communities [37, 50], women have limited input in their partners’ decisions to migrate but believe that migrating is financially beneficial for their families [38, 39, 49, 50] and that, as women, they should stick to their submissive roles in their relationships [35, 38]. However, some women are seen to break the gender roles by entering the labor market, especially by taking on the agricultural work their migrant partners may have left behind [28, 34, 38, 51]. Even though many of these women enter the workforce while their partners are away, job opportunities may be limited due to the social and cultural expectations to prioritize their motherhood role [28]. Additionally, Mexican women often remain behind to be the heads of their households, to parent on their own, and sometimes to care for aging family members [35, 38, 47, 48, 51]. It has also been discussed that if women move in to live with their relatives, they perceive receiving greater support from the family, but their lives may be under constant control, especially with regard to their purchasing power agency [33, 40]. The migrants’ infidelity while being away is also often suspected by the women who remain behind [34, 52].

### Women’s health

Health issues are found throughout the literature on Mexican women who remain behind while their partners migrate abroad. Many authors discuss mental health concerns, such as feelings of abandonment [47]; symptoms of distress (i.e. sadness, fear) [36, 39, 48]; difficulty sleeping and obsessive thinking [48]; anger [29]; anxiety, stress, and depression [32, 50, 53]; and emotional disorders [30, 36]. Many of the mental health problems have been attributed to the women’s inability to communicate with their partners [50], health emergencies [47], worries about their partners’ health and wellbeing while abroad [50], fear of their partners’ deportation [50], and the increase in the women’s roles and responsibilities (i.e. parenting on their own) [49, 50]. However, the finding of mental health problems being caused by an increase in roles and responsibilities is contradictory to other studies that found no association between the women’s mental health who remain behind and household management or family caretaking [48]. Mental health interventions have been successful in reducing depression and increasing social support among Mexican women [53], but some women prefer to ignore their concerns or simply learn to cope with the distanced relationship [37, 39]. Moreover, Mexican women are also seen to experience heart-related diseases, being overweight or obese, and higher barriers to healthcare access, like the lack of medications or healthcare practitioners [30, 50].

## Discussion

The purpose of this scoping review was to explore the scope, range, and nature of the existing literature on Mexican women who remain behind in their communities of origin while their partners migrate abroad. This review, through an intersectional lens, provided insights into the women whose voices are acknowledged in the literature and those whose voices are not. In general, most of the MWRB included in the studies had similar forms of identity as cis-heterosexual women who, in some cases, were Indigenous, and who had multiple children, were of a low socioeconomic status and possessed a low education level, and resided in Mexico’s poorest contexts. Based on the findings, I suggest seven areas of opportunities for future research and intervention.

### Gender

Mexican women who remain behind while their partners live and work in a foreign country tend to represent cis-heterosexual women living in a patriarchal context. Notably, women with different forms of identity (i.e. transgender women) were not considered in the analyzed studies. This is important as not all types of women may experience the migration phenomenon the same way. For instance, findings from this review indicate that women continue to depend on their migrant partners’ displays of hegemonic masculinity through dominance, power, and patriarchy. This is supported by research on Mexican men in the United States, where migrants often negotiate their hegemonic heterosexual masculinities by maintaining their breadwinner role within the family; these masculinities are shaped by the intersections of gender, immigration, race, and citizenship [40, 41]. Similarly, the patriarchal nature of Mexican culture can explain the migrant men’s infidelity—rooted in “machismo,” or an entitlement to patriarchy and an attribute of masculinity ideology that places men over women [42]. Thus, I suggest particular attention be paid to women who do not self-identify as cis-heterosexual, including lesbian, transgender, and bisexual women. Researching such populations would help us have a broader understanding of the experiences of remaining behind.

Most studies included women between the ages of 18 and 55, overlooking younger women. Redirecting our attention to younger populations is important given that more than 75,000 Mexican adolescents between the ages of 12 and 17 years were married in 2015 [43]. Furthermore, women without children were largely unnoticed in these studies; a majority of the women had between two to six children. Additionally, these studies failed to report if any women had children with special needs, which could have influenced their findings. This data is essential as studies on Mexican mothers of children with special needs have shown that these women suffer high levels of stress due to the caregiver burden [44].

### Social class

Most of the studies in this review focus on women from low-income backgrounds whose partners migrate to high-income countries. A recent report classified Mexico as an upper-middle-income nation; however, the Mexican States in which these investigations were conducted are among Mexico’s poorest communities [45]. The idea that most men leave Mexico to improve their family’s lifestyle is supported by research on Mexican immigrants in the United States, in which those who had immigrated reported their desire to support their families in Mexico as the primary migration driver [46]. Nonetheless, Mexican men from other states and with higher incomes migrate across transnational borders for other reasons, such as to obtain higher education or to avoid the effects of climate change and violence [55]. Hence, I can conclude that there is a lack of literature on women from wealthier families, women who reside in Mexican states with better economic growth, and women whose partners have migrated for motives other than those that are work related.

### Indigeneity

The participation of women with an Indigenous background was limited in the included studies. For instance, the voices of Indigenous women who do not speak Spanish were largely left unheard. This is problematic as more than seven million Mexicans speak at least one of the 68 Indigenous languages in the country [56]. In the face of hundreds of years of colonial oppression, the resilience of Indigenous people in Mexico has ensured their continuous survival [57]. Nonetheless, even with this resilience, intersections of gender and class continue to be challenging for Indigenous communities in Mexico, especially for Indigenous women, who are largely marginalized, underprivileged, and have low education levels [56, 57]. Based on this observation, I suggest that future research on this population seek to increase the participation of Indigenous communities. This is important as the majority of Indigenous groups in Mexico reside in states where migration rates are high.

### Health

Findings from this review mainly shed light on the mental health issues experienced by Mexican women following the departure of their migrant partners. Despite the repeated appearance of this health concern, there was only one identified intervention study that aimed to improve the women’s mental health; hence, more preventative and therapeutic interventions are needed. Moreover, women with preexisting health conditions, such as women with disabilities or women with chronic diseases (i.e. HIV, hypertension, diabetes), were missing in these studies. For example, over the last decade, most people living with HIV in Mexico have been from southern states [58], which are also classified as the poorest states and are those with the highest outgoing migration rates. Future research must address the possible interaction between remaining behind and the preexisting health conditions of women as another factor that may shape their experiences.

### Education

The education level of the women was visible across the literature. For instance, studies were mainly conducted in Mexico’s lowest education contexts, such as those within the states of Veracruz and Oaxaca. In 2018, the Mexican government announced that a higher percentage of men held a university degree compared to their female counterparts [59], and Indigenous women were also behind men in terms of completing a university degree [60]. Similarly, Indigenous women in Mexico are 30% less likely to obtain a college degree when compared to non-Indigenous women [61]. In this regard, research has shown that Mexican Indigenous college students often experience loneliness and discrimination, and this may be due to a lack of self-identity or feelings of belonging to an Indigenous group [62]. I propose that researchers and scholars consider in their research Mexican women who have a migrant partner and who live in higher-income families or in Mexican states with better financial development.

## Limitations

There are several limitations to this research. First, given the nature of scoping reviews, a formal evaluation of the quality of evidence was not conducted. Importantly, books, book chapters, and grey literature were not included in this review. Even though a comprehensive search was carried out in nine electronic databases, additional studies with alternative keywords or indexed in different databases may have been missed. Finally, studies without the Spanish or English full texts were excluded. Thus, interpretation of the findings of this scoping review must be done cautiously.

## Conclusion

This systematic scoping review informed by an intersectionality framework offers understandings into the existing empirical evidence of Mexican women who remain behind while their partners migrate abroad. Significant gaps and potential biases in the literature were discussed, and implications for future research and intervention work are proposed. These findings provide direction for future research on Mexican women who remain behind that could examine, for instance, the experiences of women who have preexisting health conditions, Indigenous women who do not speak Spanish, mothers of children with special needs, women whose partners migrated for reasons other than work, and teenage girls. I suggest that this future research work can guided by an intersectional approach. By centering our attention on the interactions among categories of identity (i.e. gender, class, race) within these women’s social, political, and cultural contexts, intersectionality can help us unveil the obscure systems of power and domination that may be shaping their experiences.

## Supporting information

S1 Checklist (PDF)
